# Flexural Behaviour of Carbon Textile-Reinforced Concrete with Prestress and Steel Fibres

**DOI:** 10.3390/polym10010098

**Published:** 2018-01-20

**Authors:** Yunxing Du, Xinying Zhang, Lingling Liu, Fen Zhou, Deju Zhu, Wei Pan

**Affiliations:** 1 Key Laboratory for Green & Advanced Civil Engineering Materials and Application Technology of Hunan Province, Changsha 410082, China; zhoufen@hnu.edu.cn (F.Z.); dzhu@hnu.edu.cn (D.Z.); 2 College of Civil Engineering, Hunan University, Changsha 410082, China; s160100086@hnu.edu.cn (X.Z.); liulingling@hnu.edu.cn (L.L.); 3 Department of Civil Engineering, The University of Hong Kong, Pokfulam, Hong Kong, China; wpan@hku.hk

**Keywords:** carbon textile-reinforced concrete, flexural behaviour, steel fibres, prestress, interfacial bonding performance

## Abstract

Four-point bending tests were adopted to investigate the influences of the number of textile layers, volume content of steel fibres, and prestress on the flexural behaviour of carbon textile-reinforced concrete (TRC). The failure mode of the specimen changed from debonding failure to shear failure, accompanied by the matrix-textile interfacial debonding with an increasing number of textile layers. The interfacial bonding performance between the textile and matrix improved with the addition of steel fibres in the TRC specimens. The presence of prestress or steel fibres improved first-crack and ultimate stresses of the TRC specimen. In comparison with the first-crack stress, a more pronounced enhancement in the ultimate stress was achieved by the addition of steel fibres. However, the effect of prestress on the first-crack stress was found to be more significant than on the ultimate stress. The prestress combined with steel fibres further improved the flexural behaviour of the TRC specimens. The prestressed TRC specimens with 1% volume content of steel fibres effectively avoided debonding. Thus, the utilization of the textiles could be improved.

## 1. Introduction

Textile-reinforced concrete (TRC) is a novel composite construction material consisting of a fine-grained concrete matrix and high-performance textile made of various fibres, such as alkali-resistant (AR) glass, carbon, or polymer fibres. The textile used in TRC is characterized by its high tensile strength and ductility. It is a good alternative for reinforcing the plain concrete. In addition, the TRC tends to be thin-walled and lightweight components due to the excellent corrosion resistance of the textile. The flexural component of the TRC has a wide prospect in structural engineering because of its beneficial mechanical properties [1,2,3,4,5].

Recently, several researchers have performed experimental and theoretical studies on the TRC flexural component [6,7,8,9]. Co-working among the filaments of the textile is significantly improved after impregnation of textiles with polymers, but the impregnated textiles are easily separated from the matrix. In this case, the stiffness of the TRC flexural component is obviously reduced, and the high tensile strength of the carbon textile is not fully exploited. Also, brittle debonding failure may occur if the longitudinal crack width is sufficiently large. An effective way to address the problems above is to improve the interfacial bonding performance between the textile and matrix. 

Several studies have indicated that the addition of short fibres in the TRC can improve the interfacial bonding performance between the textile and matrix. Barhum and Mechtcherine [10,11] studied the effects of short fibres on the fracture behavior of textile-reinforced concrete. The short fibres built new “special” adhesive cross-links which provided extra connecting points to the surrounding matrix by their random positioning on the yarn’s surface. Therefore, the bond between multifilament-yarn and the surrounding matrix improved. Pakravan et al. [12] examined the flexural behaviour of the TRC with polyvinyl alcohol (PVA) fibres using three-point bending tests. Short PVA fibres could improve the bearing capacity of the specimens and increase its crack number. Also, the failure mode of the TRC changed from single-cracking to multi-cracking. Li and Xu [13] explored the effect of the content of short PVA fibres on the flexural behaviour of TRC using four-point bending tests. The first-crack load, ultimate load and crack number of the specimens increased when the volume content of PVA fibres increased from 1% to 1.5%. The width and spacing of cracks were influenced by the bond between textile and matrix. The reduction of the crack spacing implied the improvement of bond behavior between textile and matrix. Xu et al. [14] investigated the mechanical performance of TRC under normal and high-temperature conditions using three-point bending tests. The results indicated that under normal temperature and 120 °C, the addition of polypropylene (PP) fibres greatly increased the bearing capacity of the specimens and reduced the crack width. However, the PP fibres did not have obvious effect on the bearing capacity of the specimens at 200 °C for a long time. Short fibres, especially carbon, AR glass, PP, and PVA fibres, had been used in experimental studies on TRC. Mostly, the short fibres were randomly distributed in the matrix. The present study also attempts to add short fibres in the TRC specimens. However, some parts of them are inserted into the grids of the textile, and the remaining parts are mixed into the matrix to effectively improve the interfacial bonding performance between the textile and matrix.

Prestress on the textile can delay the generation of cracks on the TRC specimens. Reinhardt et al. [15] investigated the influences of the textile type, whether the textile is impregnated or not and the prestress on the flexural behaviour of TRC using the four-point bending tests. Only a layer of textile was placed at the middle of the cross section of the specimen in these tests. The prestress on the AR glass textile improved the ultimate load and reduced the ultimate deflection of the specimens. Prestress on the carbon textile reduced the ultimate load and increased the ultimate deflection of the specimens, and the crack width at failure cannot meet the requirement for normal serviceability. However, prestress on the impregnated carbon textile increased the first-crack and ultimate loads of the specimens but reduced the ultimate deflection and crack width. Moreover, the bearing capacity was significantly improved with the increase in prestress level. Hence, the impregnated carbon textile was the most suitable for the prestressed TRC according to the results. Meyer and Vilkner [16,17] studied the flexural behaviour of the prestressed aramid TRC using the three-point bending tests. The textiles were evenly placed along the thickness of the specimens in these tests. The prestress delayed the generation of cracks and improved the post-cracking flexural stiffness and bearing capacity of the specimens but decreased the ductility. Peled [18] studied the flexural behaviour of prestressed TRC using the four-point bending tests and the interfacial bonding performance between the textile and matrix by pull out tests. The behaviour of TRC was related to textile geometry, textile type and the time at which the pre-tension was released. When the pre-tension for Kevlar textile with high modulus of elasticity was released at the seventh day after casting, the matrix-textile interfacial bonding performance, first-crack load, ultimate load and the utilization of textiles improved. Meanwhile, for the PP- and polyethylene- (PE-) knitted textile with a low modulus of elasticity, the time at which the pre-tension was released slightly affected the flexural behaviour and matrix-textile interfacial bonding performance of the specimens. At present, although many efforts have been taken on the flexural behaviour of the TRC, limited information is available on the effect of the textile arrangement. Mostly, the textiles are evenly placed along the thickness of the specimens. Hence, a new method of textile arrangement is proposed in this paper. The textiles are unsymmetrically placed along the thickness of the cross section of the TRC and mainly placed below the middle line of the cross section. Thus, the prestress on the textile can greatly improve the flexural bearing capacity and crack resistance.

The study reported in this paper aims to add steel fibres in the TRC specimens and apply prestress on the textile to improve the interfacial bonding performance between the textile and matrix and the flexural behaviour of the TRC. The four-point bending tests are employed to investigate the influences of the number of textile layers, volume contents of steel fibres and the prestress on the flexural behaviour of the carbon TRC. Moreover, the effect of the addition of steel fibres on the flexural behaviour of the prestressed TRC specimens is investigated.

## 2. Experimental Study

### 2.1. Textile Reinforcement

**Figure 1:**
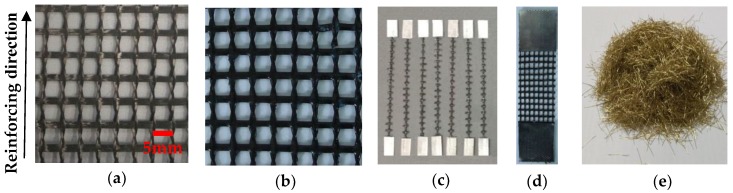
(**a**) Carbon textile; (**b**) impregnated carbon textile; (**c**) single yarn samples; (**d**) carbon textile strip; and (**e**) steel fibres.

Carbon textile (Red Nations High Performance Fiber Products Co., Ltd., Yixing, China) impregnated with epoxy resin (Goodbond, Changsha, China) was applied as internal reinforcement for TRC specimens [19,20]. The carbon textile before and after impregnation are illustrated in Figure 1a,b. The specimens in this test were one-way slabs, and the warp yarns (along the length of the textile) served as reinforcement. The cross-sectional area of single yarn was 0.218 mm^2^, calculated as the ratio of tex (the linear density of this material) to its bulk density. The mechanical properties of single yarns with 100 mm-gauge length shown in Figure 1c that cut from the impregnated textile were determined using tensile tests [21]. These tests were conducted on MTS C43.304 (MTS System Corporation, Shenzhen, China) at a rate of 2.5 mm/min. The load was measured by a 1 kN load cell at a sampling rate of 20 Hz. Mechanical properties of the carbon textile were determined by tensile tests on 40 mm × 100 mm textile strips that consisted of eight yarns, as shown in Figure 1d. The mechanical properties of single yarns and carbon textile strips are summarized in Table 1 and Table 2, respectively.

### 2.2. Steel Fibres

Steel fibres are known for their superior mechanical properties, such as high tensile strength and high modulus of elasticity, and can easily distribute in the cementitious matrix. The copper-coated chopped steel fibres (Bosaite Construction Meterials Co., Ltd., Changsha, China) used in this test are illustrated in Figure 1e, and the mechanical properties and geometric parameters of the chopped steel fibres are listed in Table 3. The density of steel fibre is obtained as the ratio of mass to its volume, and its volume is measured by drainage method.

### 2.3. Cementitious Matrix

**Figure 2:**
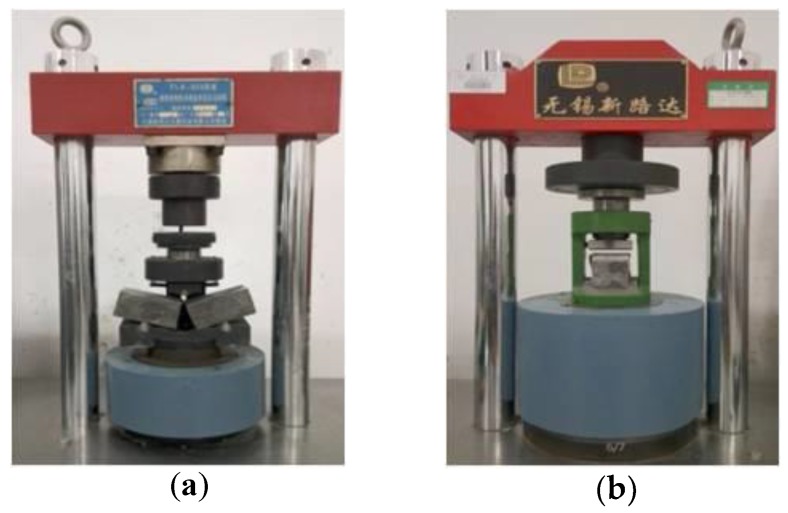
Setup of (**a**) the three-point bending and (**b**) compression test of the plain matrix.

Cementitious matrix used in TRC needs to satisfy the requirement of high workability, self-compacting property and high early strength. The maximum grain size of the aggregate was 2 mm to obtain an improved flowing capacity to ensure sufficient penetration of the matrix into the textiles. The type of cement used in the matrix is P. II 52.5 (Good Bond Construction Technic Development Co., Ltd., Changsha, China).The compressive and flexural properties of the plain cementitious matrix were obtained by the compressive and three point-bending tests. The three-point bending tests were conducted on 40 mm × 40 mm × 160 mm plain matrix specimens, as shown in Figure 2a. Then, the compressive tests were conducted on the broken prismatic specimens from the bending tests, as shown in Figure 2b. The free flow test of the matrix was performed according to Chinese Standard GB/T 2419-2016 (Test method for fluidity of cement mortar). The mixture and properties of the cementitious matrix are summarized in Table 4 and Table 5, respectively. The fly ash, silica fume, slag, sand and superplasticizer used in the cementitious matrix purchased from Good Bond Construction Technic Development Co., Ltd. (Changsha, China).

### 2.4. Specimen Manufacturing

**Figure 3:**
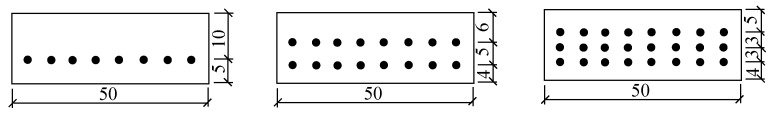
Cross section of the specimens with different number of textile layers (unit: mm).

**Figure 4:**
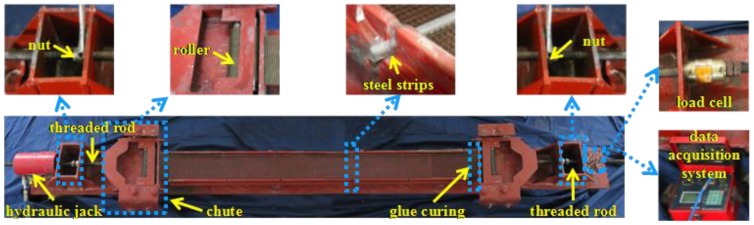
Illustration of prestress tensioning device.

**Figure 5:**
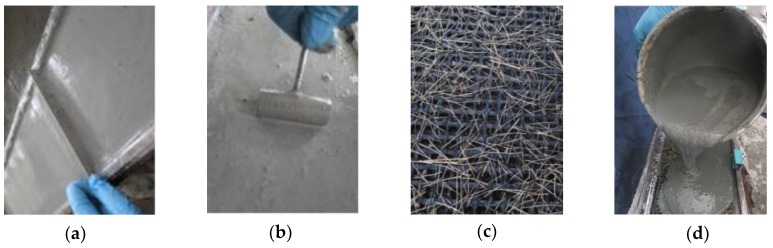
Preparation of the plates: (**a**) levelling the surface; (**b**) smoothing the surface; (**c**) steel fibres inserted in the grids; and (**d**) steel fibres mixed with the matrix.

The thickness of all the TRC plates was 15 mm. One to three layers of textile were placed along the thickness of the plates, as illustrated in Figure 3. The manufacturing process started with all the textiles stretching and fixing evenly to the prestress tensioning device, as depicted in Figure 4. For the prestressed plates, the pre-tension was applied to the textiles using the tensioning system (Figure 4). The two ends of the textiles were coated by glue to reduce the loss of prestress force resulting from the relaxation of prestressed textiles. The tensioning state should be maintained for 24 h. Then, tension was reapplied on the textiles to the prescribed value according to the corresponding pre-tension loss [22]. Subsequently, the cementitious matrix was cast into the mould and was fully vibrated by a portable surface concrete vibrator to allow its penetration through the grids of the textiles and simultaneously reduce the pore of the plates. Finally, the surface of the plates should be levelled and smoothed, as shown in Figure 5a,b.

The plates were covered with wet clothes and foil after the cementitious matrix was initially set. The prestressed and non-prestressed TRC plates were allowed to harden for 7 and 2 days, respectively. The force recorded by the acquisition system at the time of demoulding for the prestressed TRC plates was considered the actual control prestress force (*F*_con_) applied on the textiles. Then, all the plates were demoulded and cured at 20 °C with 95% relative humidity until testing was performed after 28 days. During the preparation of the plates with steel fibres, a part of the steel fibres was vertically or obliquely inserted into the grids of the textiles, and the remaining steel fibres were evenly mixed into the cementitious matrix, as depicted in Figure 5c,d. In addition, all the specimens had the same dimension of 295 mm × 50 mm × 15 mm. They were cut from large plates using a diamond saw before the tests to reduce scattering in the experimental results. At least three specimens were tested for each kind of experimental case. A thin layer of white paint was sprayed on the surfaces of the specimens for convenient observation of the crack development.

### 2.5. Four-Point Bending Test Setup

**Figure 6:**
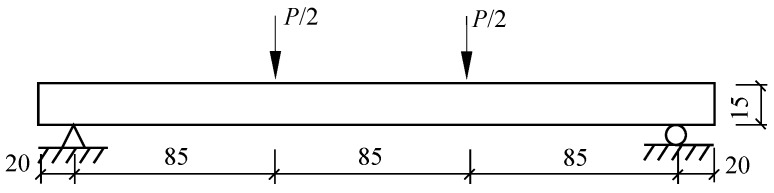
Schematic setup of the four-point bending test.

Four-point bending tests were performed on MTS C43.304 (MTS System Corporation, Shenzhen, China) under displacement control with a constant rate of 0.5 mm/min. The loading diagram is presented in Figure 6. The load was measured by the load cell, and the deflection was measured by a 100-millimeter clip-on extensometer (Sanjing Testing Instrument Co., Ltd., Changchun, China) attached in the midspan. The loads and deformations were recorded simultaneously at a sampling rate of 20 Hz through the computer connected to the testing machine. The typical results of the experiment are described as load versus midspan deflection curves (load–deflection curve). The flexural nominal stress is calculated using Equation (1) below:

(1) $$\sigma =\frac{P\cdot l}{b\cdot h^{2}},$$

where σ is the flexural nominal stress, *P* is the load, *l* is the span of the specimen, *b* is the width of the cross section and *h* is the height of the cross section.

### 2.6. Experimental Scheme

In this test, the influences of the number of textile layers, volume contents of steel fibres and prestress on the flexural behaviour of carbon TRC were investigated. Firstly, the specimens with one to three layers of textile were tested to study the influence of the number of textile layers on the flexural behaviour of TRC specimens. Secondly, specimens without textile but with 1% volume content of steel fibres and those with one to three layers of textile and 1% volume content of steel fibres were tested to study the influence of the steel fibres on the flexural behaviour of TRC specimens. Thirdly, specimens with one layer of textile and different volume contents (0%, 0.5%, 1%, and 2%) of steel fibres were tested to study the influence of volume contents of steel fibres on the flexural behaviour of TRC specimens. Finally, specimens with one to three layers of textile and a prestress level of 15% on textiles were tested to study the influence of prestress on the flexural behaviour of TRC specimens. The prestress level is a ratio of the control prestress force (*F*_con_) to the total tensile bearing capacity (*F*_t_) of the textile, where *F*_t_ is the product of the tensile bearing capacity of a single layer of textile and the number of textile layers. Meanwhile, the influence of the prestress and 1% volume content of steel fibres on the flexural behaviour of the TRC specimens was investigated. The rules of labelling the experimental cases were as follows: P represents the prestress level applied on the textile, C represents the number of textile layers, and S represents the volume content of the steel fibres. For example, P15C3S1 represented a TRC specimen with a prestress level of 15%, three layers of textile and a steel fibre volume content of 1%.

## 3. Experimental Results and Discussion

### 3.1. Effect of the Number of Textile Layers

**Figure 7:**
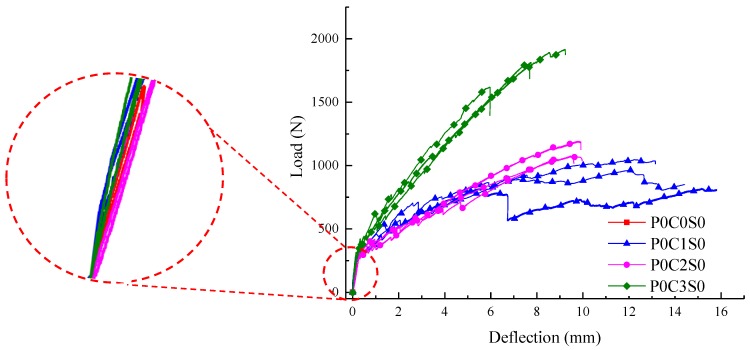
Load-deflection curves of the TRC specimens with different number of textile layers.

**Figure 8:**
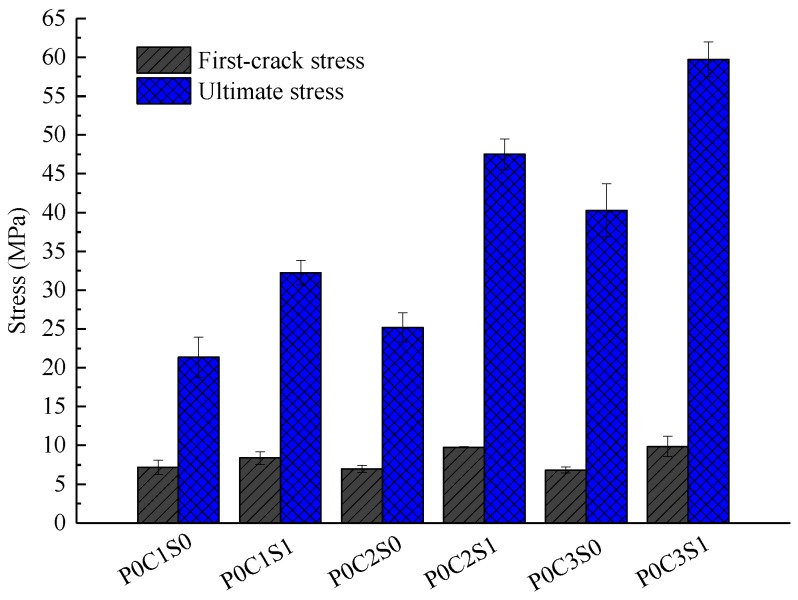
First-crack and ultimate stresses of the TRC specimens with different number of textile layers and addition of 1% volume content of steel fibres.

The influence of different textile layers on the flexural behaviour of TRC specimens is presented through the load–deflection curves in Figure 7. In Figure 8, the first-crack and ultimate stresses of the specimens are compared. The experimental data, including the first-crack load, first-crack stress, ultimate load, ultimate stress, ultimate deflection, flexural toughness and crack number, of all the TRC specimens are listed in Table 6. Toughness, which is an important index for presenting the energy absorption capability of the TRC specimens, is calculated as the area under the load–deflection curve.

The load increased linearly with the deflection for the un-cracked specimens, as shown in Figure 7. After cracking, the curves showed considerable difference between unreinforced and reinforced specimens. For the unreinforced specimens, the load suddenly dropped after the first crack appeared, and the specimens showed a brittle failure with a single crack. For the TRC specimen, the load-deflection curve started to fluctuate after the first crack appeared, and the load continued to increase after a course of fluctuation. Finally, failure of the TRC specimen occurred after reaching the ultimate load.

The bearing capacity of the TRC specimens gradually improved with increased textile layers, as displayed in Figure 7 and Figure 8 and Table 6. The average ultimate stress of one-layer TRC specimens was 21.35 MPa. The average ultimate stress of two-layer and three-layer TRC specimens increased by approximately 17.6% and 88.7%, respectively, compared with the one-layer TRC specimens. The two-layer TRC specimens failed in debonding along the matrix-textile interface, and the tensile strength of the textiles was not utilized. However, the three-layer TRC specimens showed shear failure mode. Therefore, the ultimate stress of the three-layer TRC specimens was greatly improved.

In Figure 7, the slope of the curve in the post-cracking stage was lower than that in the pre-cracking stage, which indicated that the flexural stiffness of the cracked TRC specimen reduced. However, smaller reduction in the flexural stiffness could be observed with the increase in the textile layers. In the post-cracking stage, the crack width increased and cracks propagated upward as the load increased. Thus, the neutral axis of the TRC specimen moved upward, and the contribution of the textiles on the section stiffness becomes more noticeable because the textiles mainly bore the tension force at the cracks. Therefore, smaller reduction in post-cracking flexural stiffness of the TRC specimens could be observed with increased textile layers [23]. Simultaneously, the deformation of the specimen under the same load was reduced with increasing number of textile layers.

**Figure 9:**
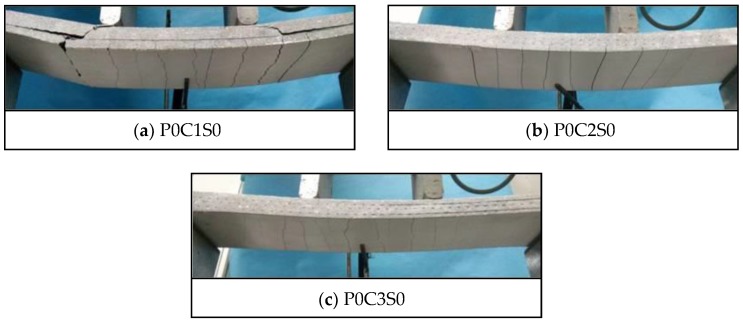
Crack patterns of the TRC specimens with different number of textile layers.

**Figure 10:**
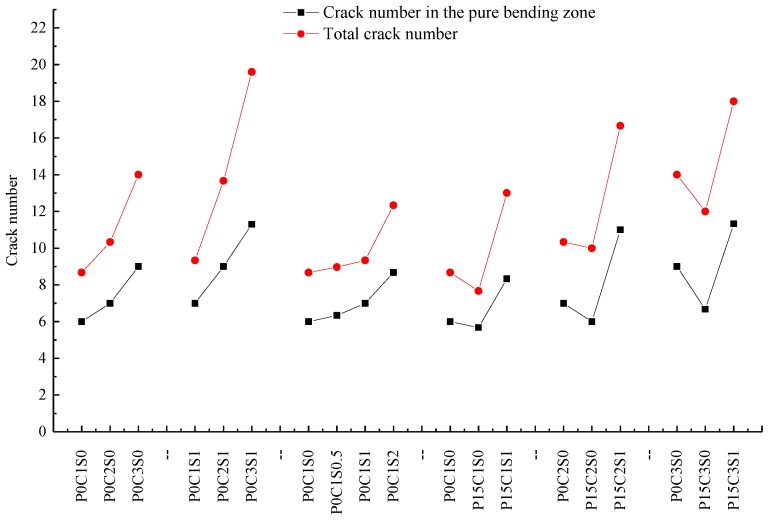
Crack number of the TRC specimens.

Figure 9 exhibits the crack patterns of P0C1S0, P0C2S0, and P0C3S0. The crack numbers in the tension and pure bending zones of all the specimens were compared, as shown in Figure 10. The TRC specimens showed multiple cracking behaviour under loading. The cracks were uniformly distributed in the tension zone of the specimens, and most of them appeared in the pure bending zone of the specimens. When the cracking moment of the weakest section was reached, the first crack appeared at the corresponding location of the TRC specimen. As the load continued to increase, another crack appeared at the next weak section of the specimen. Repetition of this process led to the phenomenon of multiple cracking of the TRC specimens. In general, increased textile layers result in increased crack number but reduced average crack spacing, as shown in Figure 10 and Table 6.

**Figure 11:**
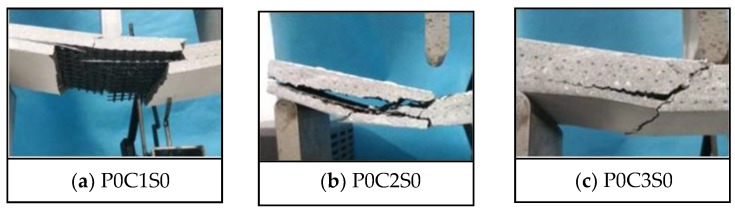
Failure modes of the TRC specimens with different number of textile layers.

**Figure 12:**
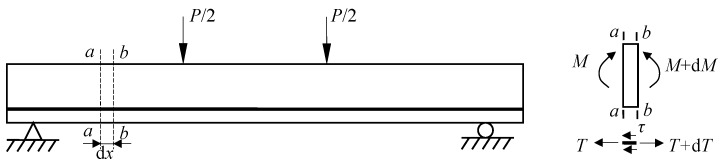
Force on the infinitesimal segment in the shear-bending zone and textile.

Figure 11 exhibits the failure modes of P0C1S0, P0C2S0, and P0C3S0. The failure of P0C1S0 and P0C2S0 resulted from the longitudinal crack along the matrix-textile interface, and the longitudinal crack propagated with the increased load. Finally, P0C1S0 and P0C2S0 broke down due to the collapse of the matrix, as shown in Figure 11a,b. However, P0C3S0 demonstrated the typical shear failure accompanied by slight matrix-textile interfacial debonding, as shown in Figure 11c. The possibility of debonding failure in the TRC specimen declined with increasing number of textile layers. As demonstrated in Figure 12 , an infinitesimal segment of length d*x* was taken from the shear-bending zone of the TRC specimens. A tension increment d*T* of the textile between the *b*-*b* section and *a*-*a* section was apparent because the moment in the *b*-*b* section was greater than that in the *a*-*a* section. The interfacial stress on the textile along the infinitesimal segment of length d*x* could balance the tension increment d*T*. The textiles were separated from the matrix when the interfacial stress exceeded the bond strength of the matrix-textile interface. However, under the same load, the tensile stress of the textile was reduced with increasing number of textile layers. Thus, the tension increment d*T* of the textile along the infinitesimal segment of length d*x* was reduced, as well as the matrix-textile interfacial stress. The debonding length along the matrix-textile interface was consequently shortened with increasing number of textile layers.

### 3.2. Effect of Steel Fibres

**Figure 13:**
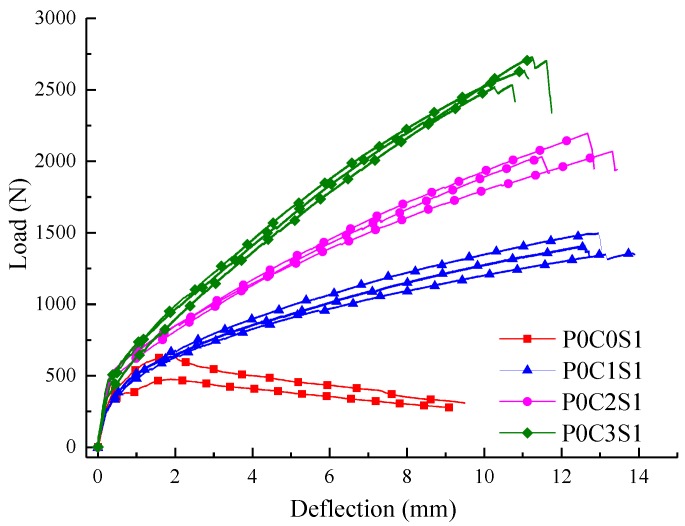
Load-deflection curves of the TRC specimens with 1% volume content of steel fibres.

Figure 13 shows the load-deflection responses of the specimens P0C1S1, P0C2S1, and P0C3S1. The first-crack and ultimate stresses of the specimens are compared in Figure 8. In Figure 13, the load of specimen P0C0S1 slightly decreased after the ultimate load, and 2–3 cracks could be observed at failure of the specimen. This phenomenon indicated that the ductility of the plain matrix was improved by steel fibres. In Figure 8 and Table 6, the ultimate stress of P0C0S1 was 12.54 MPa, which increased by 151.3% in comparison with the plain matrix. In comparison with those of P0C1S0, the first-crack stress, ultimate stress and toughness of P0C1S1 increased by 16.7%, 51.1%, and 23.5%, respectively. The first-crack stress, ultimate stress and toughness of P0C2S1 increased by 39.8%, 89.2%, and 143.1%, respectively, compared with those of P0C2S0. The first-crack stress, ultimate stress and toughness of P0C3S1 increased by 44.3%, 48.3%, and 119.1%, respectively, compared with those of P0C3S0. The steel fibres in the matrix improved the crack resistance of the specimens, and as a result, the first-crack stress was increased. In addition, the steel fibres inserted into the grids of the textiles improved the interfacial bonding performance between the textile and matrix, and as a result, the ultimate stress and flexural toughness were increased. With increased number of textile layers, the influence of the steel fibres on first-crack stress and flexural toughness became more pronounced. However, the influence of the steel fibres on the ultimate stress of the two-layer specimen was most significant compared with that of the one-layer and three-layer specimens. The two-layer TRC specimens without steel fibres showed serious debonding failure, and the tensile strength of the textile was not utilized. However, the matrix-textile interfacial bonding performance of the two-layer specimens was improved due to the steel fibres, thereby providing a better utilization of the textiles. Therefore, the ultimate stress of the two-layer specimens were significantly improved.

**Figure 14:**
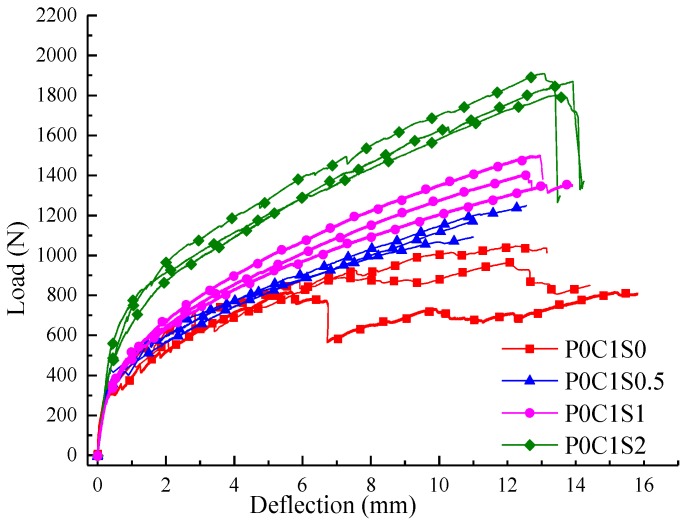
Load-deflection curves of the TRC specimens with different volume contents of steel fibres.

**Figure 15:**
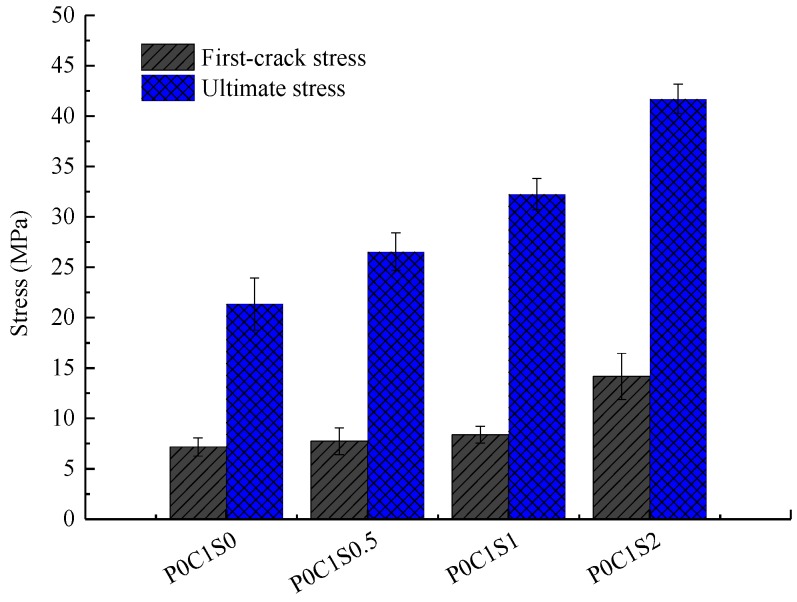
First-crack and ultimate stresses of the TRC specimens with different volume contents of steel fibres.

Figure 14 shows the load-deflection responses of the TRC specimens with single layer of textile and 0.5%, 1%, and 2% volume content of steel fibres. In Figure 15, the first-crack and ultimate stresses of the corresponding specimens were compared. In Figure 14 and Figure 15 and Table 6, the first-crack stress of the specimens with 0.5%, 1%, and 2% volume content of steel fibres increased by 7.8%, 16.7%, and 84.7%, respectively, compared with those of P0C1S0. The increments in ultimate stress for the specimens with 0.5%, 1%, and 2% volume content of steel fibres were 24.3%, 51%, and 95.4%, respectively, compared with those of P0C1S0. A 0.5% volume content of steel fibres did not show considerable effect on the flexural toughness of the specimen. However, the flexural toughness of the specimens with 1% and 2% volume content of steel fibres increased by 23.5% and 73.5%, respectively, compared with those of P0C1S0. Within the scope of the test, the first-crack stress and ultimate stress improved with increasing volume content of steel fibres, and improvement in the ultimate stress was greater than that in the first-crack stress. When the volume content of steel fibres is 0.5%, the defects in the matrix due to the addition of steel fibres decrease the ultimate deflection of the specimens, thus no improvement on the flexural toughness of the specimens with 0.5% volume content of steel fibres can be observed. Moreover, the load-deflection curve became smoother with the increase in the steel fibres in the specimen.

**Figure 16:**
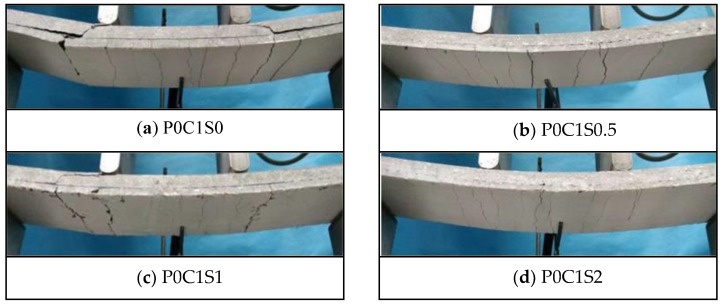
Crack patterns of the TRC specimens with different volume contents of steel fibres.

Figure 16 demonstrates the cracking patterns of specimens P0C1S0, P0C1S0.5, P0C1S1, and P0C1S2. The steel fibres could effectively increase the crack number of the specimens and reduce the average crack spacing accordingly, as displayed in Figure 12 and Figure 16 and Table 6. The cracks at the bottom of P0C1S0 and P0C1S0.5 were straight continuous cracks, and the cracks at the bottom of P0C1S1 and P0C1S2 were irregular short cracks. Increasing the volume content of steel fibres resulted in its increased restraint to matrix, changing the cracking pattern at the bottom of the specimens. The bridging effect of the steel fibres considerably restrained the propagation of cracks. However, the ultimate deflection of the TRC specimens with more steel fibres increased, which led to the increase in the total width of the cracks at the bottom of the specimens. As a result, the crack number was increased, and the average crack spacing was reduced.

**Figure 17:**
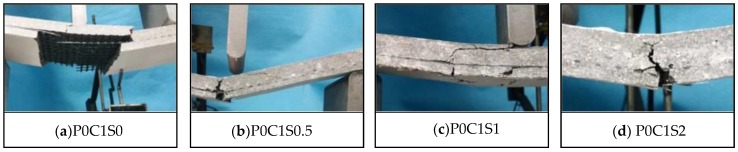
Failure modes of the TRC specimens with different volume contents of steel fibres.

The failure modes of the TRC specimens with different volume contents of steel fibres are shown in Figure 17. When P0C1S2 failed, the bottom textile was broken, and debonding along the matrix-textile interface did not occur, as depicted in Figure 17d. The 2% volume content of steel fibres in the specimens prevented the matrix-textile interfacial debonding. Meanwhile, no obvious oblique crack could be observed on P0C1S2, which indicated that the shear behaviour of the specimen improved. In Figure 17b,c, the P0C1S0.5 and P0C1S1 failed along with matrix-textile interfacial debonding, and the failure mode of P0C1S1 was shear failure. The failure modes of P0C1S0.5 and P0C1S1 indicated that the 0.5% and 1% volume contents of steel fibres could not adequately improve the matrix-textile interfacial bonding performance and shear behaviour.

**Figure 18:**
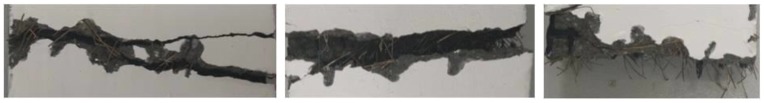
Bridging effect of the steel fibres at the cracks.

In general, the steel fibres improved the bearing capacity and flexural toughness of the TRC specimens. The cracking pattern, which featured multiple-cracking behaviour, could be observed on the specimens with steel fibres. The steel fibres that bridged over the cracks (Figure 18) were pulled out with the increase in crack width. The process of pulling out the steel fibres consumed energy, resulting in the improvement of the flexural toughness. Simultaneously, the steel fibres that bridged over the cracks bore the tensile stress transferred from the cracking matrix, thus improving the bearing capacity of the specimens. The anchoring effect of the steel fibres inserted in the grids of the textiles prevented relative slip between the textiles and matrix, resulting in the improvement of the matrix-textile interfacial bonding performance. Improved interfacial bonding performance could ensure co-working between the textile and matrix and better use of the tensile strength of the textiles. As a result, the bearing capacities of the TRC specimens were improved. Moreover, the crack number was increased, average crack spacing was reduced and the load-deflection curves became smooth with increased volume of steel fibres.

### 3.3. Effect of Prestress

**Figure 19:**
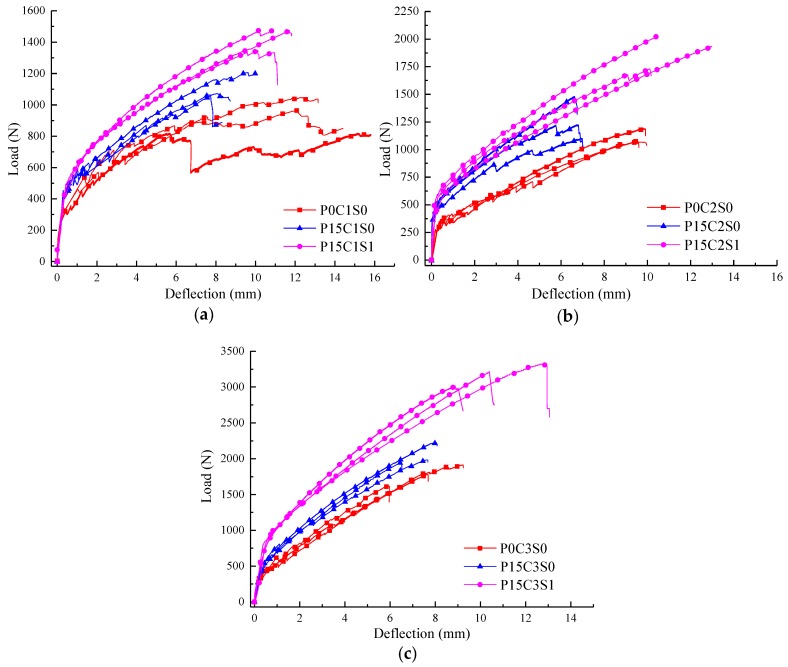
Load-deflection curves of the (**a**) one-layer, (**b**) two-layer and (**c**) three-layer prestressed TRC specimens with 0% and 1% volume contents of steel fibres.

**Figure 20:**
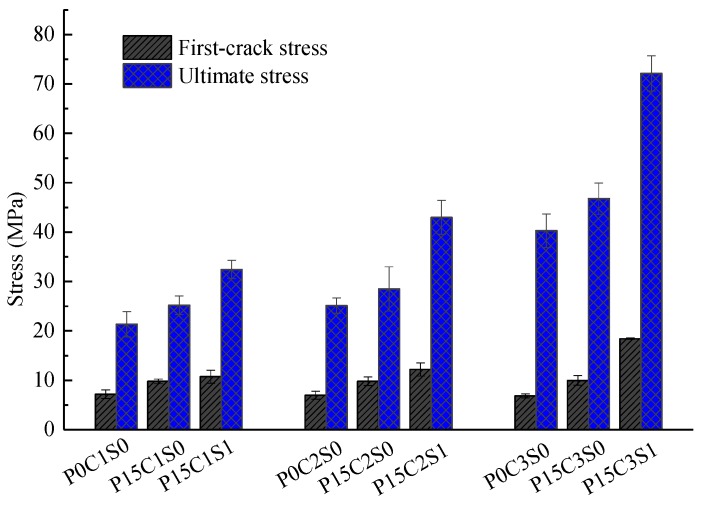
First-crack and ultimate stresses of the TRC specimens with prestress and steel fibres.

In this section, the influence of 15% prestress level on the flexural behaviour of the TRC specimens, and the influence of 1% volume content of steel fibres on the flexural behaviour of TRC specimens with 15% prestress level are discussed. Figure 19 and Table 6 summarized the experimental data of the prestressed TRC specimens. In Figure 20, the first-crack and ultimate stresses of the corresponding specimens were compared. As shown in Figure 19, prestress on the textile improved the bearing capacity of the TRC specimens and reduced the ultimate deflection. However, the bearing capacity of the prestressed TRC specimens was further improved, and the deforming capacity was enhanced due to the addition of steel fibres. In Figure 20 and Table 6, the first-crack stresses of P15C1S0 and P15C1S1 increased by 36.2% and 49.4%, respectively, compared with those of P0C1S0, and the increases in the ultimate stress were 18% and 51.9%. The first-crack stresses of P15C2S0 and P15C2S1 increased by 40.5% and 74.8%, respectively, compared with those of P0C2S0, and the corresponding increases in the ultimate stress were 13.4% and 71.2%. The first-crack stresses of P15C3S0 and P15C3S1 increased by 45.9% and 169.4%, respectively, compared with those of P0C3S0, and the corresponding increases in the ultimate stress were 16.2% and 79.1%.

**Figure 21:**
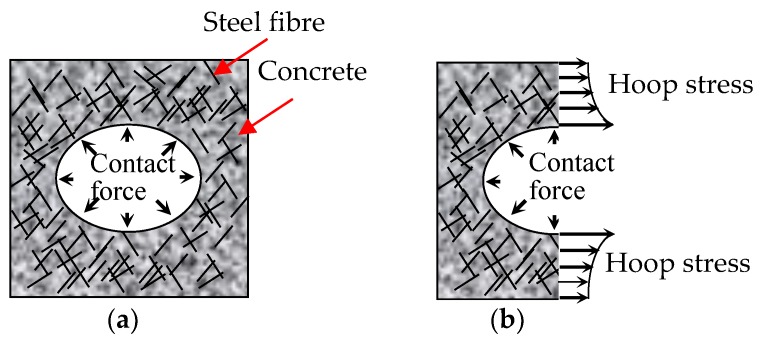
Diagram of (**a**) force in the interface between yarns and matrix and (**b**) stress of the cementitious matrix after releasing the pre-tension on textile.

The comparison of data above indicated that the prestress on textile improved the first-crack and ultimate stresses of the TRC specimens, and the improvement on the first-crack stress was more pronounced. The release of the pre-tension on both ends of the textile provided the matrix initial compressive stress during the manufacture of the prestressed TRC plates, and the initial compressive stress on the matrix should be offset before cracking. Thus, the first-crack stress was increased by the prestress. In addition, the prestress brought about the effects of Poisson’s ratio. The warp yarns possessed a certain retraction after the release of the pre-tension on the textile. The cross section of yarns increases due to effect of Poisson’s ratio, thus the warp yarns squeezes the surrounding cementitious matrix, as depicted in Figure 21a. Therefore, the interfacial friction, that is, bond performance, between the textile and matrix improved, and the tensile strength of the textile was better used [24,25]. However, prestress reduced the deforming capacity of the TRC specimens, thereby reducing the ultimate deflection. For a prestressed specimen, the textile obtained a certain initial strain before testing. The ultimate tensile strain of the textile was constant. Hence, the maximal deformation of the bottom textile decreased due to the initial strain of the textile, thereby reducing the ultimate deflection of the TRC specimens. The increase in the first-crack stress due to prestress was particularly pronounced with the increase in textile layers. The initial compressive stress on the matrix increased with more textile layers in the specimens, hence higher tensile stress was required. Adding 1% volume content of steel fibres into the prestressed TRC specimens could further improve the first-crack and ultimate stresses. Table 5 showed that the effect of the prestress on the first-crack stress was more pronounced than that on the ultimate stress, and the effect of the steel fibres on the ultimate stress was more pronounced than that on the first-crack stress.

**Figure 22:**
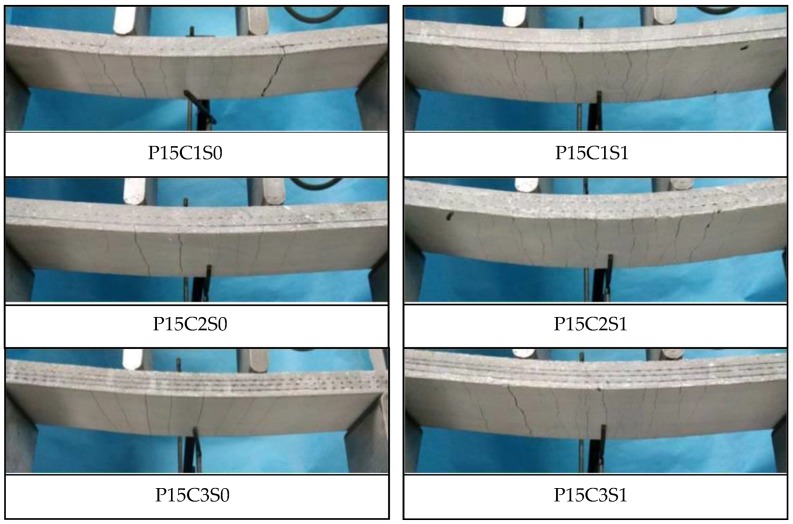
Crack patterns of the prestressed TRC specimens.

Figure 22 demonstrates the cracking patterns of the prestressed TRC specimens. The specimens exhibited multiple cracking behaviour, but the crack numbers of P15C1S0, P15C2S0, and P15C3S0 were less than those of the non-prestressed specimens. With regard to P15C1S1, P15C2S1, and P15C3S1, the prestress combined with steel fibres further improved the interfacial bonding performance between the textile and matrix. In addition, the steel fibres considerably restrained the propagation of cracks and increased the ultimate deflection of the specimens. Therefore, the crack number of the specimens increased and the average crack spacing was reduced.

**Figure 23:**
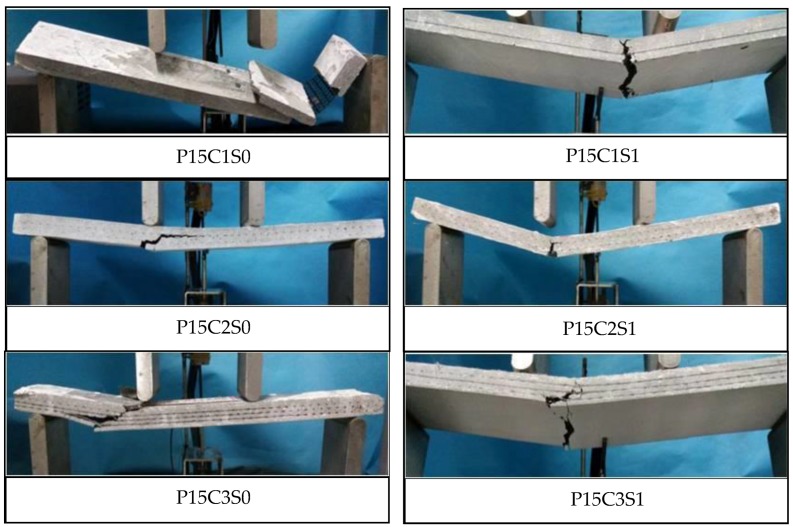
Failure modes of the prestressed TRC specimens.

The failure modes of the prestressed TRC specimens are shown in Figure 23. Although the prestress on the textiles could improve the matrix-textile interfacial bonding performance of P15C1S0, P15C2S0, and P15C3S0, the failure modes of the prestressed specimens were similar with those of the non-prestressed ones. The bottom textile was broken at failure for P15C1S1, P15C2S1, and P15C3S1, and debonding in the matrix-textile interface and oblique cracks in the shear-bending zone could not be observed. The matrix with steel fibres could better bear the circumferential stress caused by the transversal expansion of the warp yarns, as depicted in Figure 21b. Therefore, the friction between the textile and matrix were effectively improved. The failure modes above indicated that the steel fibres could further improve the matrix-textile interfacial bonding performance, leading to the enhancement on the flexural and shear behaviour of the TRC specimens.

## 4. Conclusions

In this paper, the influences of the number of textile layers, volume content of the steel fibres, and prestress on the flexural behaviour of carbon TRC are investigated using four-point bending tests. With the increase in the number of textile layers, a significant improvement on the bearing capacity of the specimens and a smaller reduction in the flexural stiffness of the cracked specimens were observed; in addition, the failure mode of the specimen changed from debonding failure to shear failure accompanied by the matrix-textile interfacial debonding. Although the prestress on the textiles was found to improve the interfacial bonding performance between the textile and matrix, the failure modes of the prestressed specimens were similar with the non-prestressed ones; thus, the tensile strength of the textiles was not fully utilized. The steel fibres improved the interfacial bonding performance between the textile and matrix and the shear behaviour of the TRC specimens; thus the bearing capacity and flexural toughness of the specimens were improved. The crack number of the specimens increased but the average crack spacing reduced with the increasing volume content of steel fibres. The presence of prestress or steel fibres improved both first-crack and ultimate stresses of the TRC specimen. The effect of steel fibres on the ultimate stress was more significant than that on the first-crack stress. However, the effect of prestress on the first-crack stress was more significant than that on the ultimate stress. For the non-prestressed specimens with 2% volume content of steel fibres and the prestressed specimens with 1% volume content of steel fibres, the bottom textile was broken at failure, and no debonding in the matrix-textile interface could be observed.

## Figures and Tables

**Table 1:** Mechanical properties of carbon yarns.

Roving specification	Tensile strength (MPa)	Young’s modulus (GPa)	Ultimate strain (%)	Density (g/cm³)	Cross-sectional area (mm²)
6 K	2484	138	1.8	1.8	0.218

**Table 2:** Mechanical properties of carbon textile strip.

Strip size	Tensile load bearing capacity (kN)	Tensile strength (MPa)	Ultimate strain (%)
100 mm × 40 mm	4	2293.58	1.67

**Table 3:** Properties of steel fibres.

Diameter (mm)	Length (mm)	Density (g/cm³)	Tensile strength (MPa)	Young’s modulus (GPa)
0.18–0.23	12–15	8.5	2850	200

**Table 4:** Mixture of the cementitious matrix.

Material	Cement	Fly ash	Silica fume	Slag	Sand	Superplasticizer	Water
Content (kg/m^3^)	800	100	50	50	1200	2	286

**Table 5:** Properties of the cementitious matrix.

Days	Compressive strength (MPa)	Flexural strength (MPa)	Flowability	
			Initial value	Retention value (30 min)
7	62.5	11.5	340 mm	320 mm
28	76.7	12.3		

**Table 6:** Flexural behaviour of the textile-reinforced concrete (TRC) specimens with different number of textile layers, volume contents of steel fibres, and prestress.

Specimen	Average value (standard deviation)								Failure mode
	First-crack load (N)	First-crack stress (MPa)	Ultimate load (N)	Ultimate stress (MPa)	Ultimate deflection (mm)	Flexural toughness (N·mm)	Crack number in the pure bending zone	Total crack number	
P0C0S0	220.00(10.51)	4.99(0.83)	220.00(10.51)	4.99(0.83)	0.17(0.001)	21(4)	1.00(0.00)	1.00(0.00)	F
P0C1S0	317.01(40.19)	7.18(0.91)	941.96(114.35)	21.35(2.59)	9.97(3.74)	10,775(473)	6.00(1.00)	8.67 (0.58)	D
P0C2S0	307.74(36.87)	6.98(0.83)	1108.07(69.75)	25.12(1.58)	9.77(0.15)	7281(445)	7.00(1.72)	10.33(3.21)	D
P0C3S0	301.59(17.27)	6.84(0.39)	1777.28(150.71)	40.28(3.42)	7.60(1.64)	8592(2599)	9.00(3.00)	14.00(4.58)	S + D
P0C0S1	372.56(1.85)	8.44(1.52)	553.24(112.01)	12.54(2.54)	1.76(0.003)	3849(647)	2.50(0.71)	2.50(0.71)	F
P0C1S1	369.87(36.51)	8.38(0.83)	1422.70(68.44)	32.25(1.55)	13.08(0.62)	13,309(670)	7.00(0.00)	9.33 (0.58)	F + S
P0C2S1	430.63(29.31)	9.76 (0.66)	2096.86(86.88)	47.53(1.97)	12.47(0.92)	17,699(1768)	9.00(0.00)	13.67(1.53)	S + D
P0C3S1	435.35(56.95)	9.87(1.29)	2635.76(98.24)	59.74(2.23)	10.98(0.25)	18,821(1552)	11.33(3.06)	19.67(5.77)	F
P0C1S0.5	341.32(58.85)	7.74(1.33)	1171.02(81.97)	26.54(1.86)	11.51(0.97)	10,146(2066)	6.33(1.53)	8.97(0.58)	F + D or D
P0C1S1	369.87(36.51)	8.38(0.83)	1422.70(68.44)	32.25(1.55)	13.08(0.62)	13,309(670)	7.00(0.00)	9.33 (0.58)	S + D
P0C1S2	585.24(101.81)	13.26(2.30)	1840.33(64.32)	41.71(1.46)	13.51(0.47)	18,695(251)	8.67(0.58)	12.33(0.58)	F
P15C1S0	431.56(20.26)	9.78(0.46)	1111.34(82.64)	25.19(1.87)	8.47(1.02)	7437(1373)	5.67(2.53)	7.67 (2.52)	D
P15C1S1	473.33(57.26)	10.73(1.30)	1430.37(81.85)	32.42(1.86)	11.15(0.48)	11,841(617)	8.33(1.15)	13.00(2.00)	F
P15C2S0	433.05(38.31)	9.81(0.87)	1257.17(197.78)	28.49(4.48)	6.50(0.13)	6175(673)	6.00(1.00)	10.00(2.00)	D
P15C2S1	538.35(58.18)	12.20(1.32)	1897.59(151.62)	43.01(3.44)	11.17(1.56)	14,600(2199)	11.00(1.73)	16.67(1.53)	F
P15C3S0	440.47(43.79)	9.98(0.99)	2064.92(139.33)	46.80(3.16)	7.39(0.82)	9953(1794)	6.67(1.53)	12.00(3.00)	S + D
P15C3S1	813.18(5.91)	18.43(0.13)	3182.86(158.05)	72.14(3.58)	10.65(1.97)	23,412(5340)	11.33(1.53)	18.00(1.00)	F

Note: F means flexural failure; D means debonding failure; F + D means flexural failure with debonding; S + D means shear failure with debonding.
